# Clinical and laboratory prognosticators of atrophic papulosis (Degos disease): a systematic review

**DOI:** 10.1186/s13023-021-01819-z

**Published:** 2021-05-06

**Authors:** Justin D. Lu, Muskaan Sachdeva, Orli M. Silverberg, Lee Shapiro, David Croitoru, Rebecca Levy

**Affiliations:** 1 Michael G. DeGroote School of Medicine, McMaster University, Hamilton, ON Canada; 2 Faculty of Medicine, University of Toronto, Toronto, ON Canada; 3 Department of Medicine, Division of Rheumatology, Albany Medical College, Albany, NY USA; 4 Division of Dermatology, Department of Medicine, Women’s College Hospital, 76 Grenville St, 3rd Floor, Toronto, ON M5S 1B2 Canada; 5 Department of Dermatology, The Hospital for Sick Children, Toronto, ON Canada; 6 Department of Pediatrics, University of Toronto, Toronto, ON Canada

**Keywords:** Degos disease, Malignant atrophic papulosis, Benign atrophic papulosis, Inflammatory cytokines

## Abstract

**Background:**

Degos disease is a rare vascular disorder with a cutaneous-limited form, benign atrophic papulosis (BAP), and a systemic variant, malignant atrophic papulosis (MAP). Despite the poor prognosis of MAP, no study has established features associated with systemic disease.

**Objectives:**

The aims of this systematic review were to: (1) summarize clinical features and treatments implemented for patients with MAP and BAP (2) identify clinical and laboratory factors associated with the development of MAP, compared to BAP.

**Methods:**

We systematically searched MEDLINE and Embase from inception to April 2020. Demographic and clinical features of Degos patients were presented descriptively; multivariable logistic regression was performed to identify associations with MAP.

**Results:**

We identified 99 case studies, comprising 105 patients. MAP (64%) had a 2.15 year median survival time from cutaneous onset, most often with gastrointestinal or central nervous system involvement. We found that elevations in either of erythrocyte sedimentation rate (ESR) or C-reactive protein (CRP) were associated with systemic involvement (OR 2.27, *p* = 0.023). Degos secondary to an autoimmune connective tissue disease was found to be inversely associated with MAP (OR 0.08, *p* = 0.048).

**Conclusions:**

Elevated ESR or CRP is associated with MAP and may be a predictor of systemic involvement for patients with Degos disease. In addition, secondary Degos disease is associated with a favourable prognosis. Clinicians should be aware of the differences between primary and secondary Degos and the utility of ESR or CRP in identifying disease evolution to systemic involvement. The utility of ESR and CRP to identify systemic involvement should be further explored.

**Supplementary Information:**

The online version contains supplementary material available at 10.1186/s13023-021-01819-z.

## Introduction

Degos Disease, also known as Köhlmeier-Degos disease, was first identified in 1941 and subsequently described as a unique thrombotic microangiopathic entity in 1942 by Degos et al. [1]. Two subtypes have been described, malignant atrophic papulosis (MAP) and the cutaneous-limited form, benign atrophic papulosis (BAP). The former results in universal mortality with no treatment consistently effective in disrupting the natural history [1]. There have been approximately 200 to 300 cases of atrophic papulosis described in the literature to date [2], and the etiology of the disease remains unknown. Reports of familial clusters of the disease exist, with increased incidence in first-degree relatives, suggests a possible genetic contribution [3–5]. Suggested pathogenetic mechanisms include vasculitis, coagulopathy, and primary dysfunction of endothelial cells [6].

**Fig. 1:**
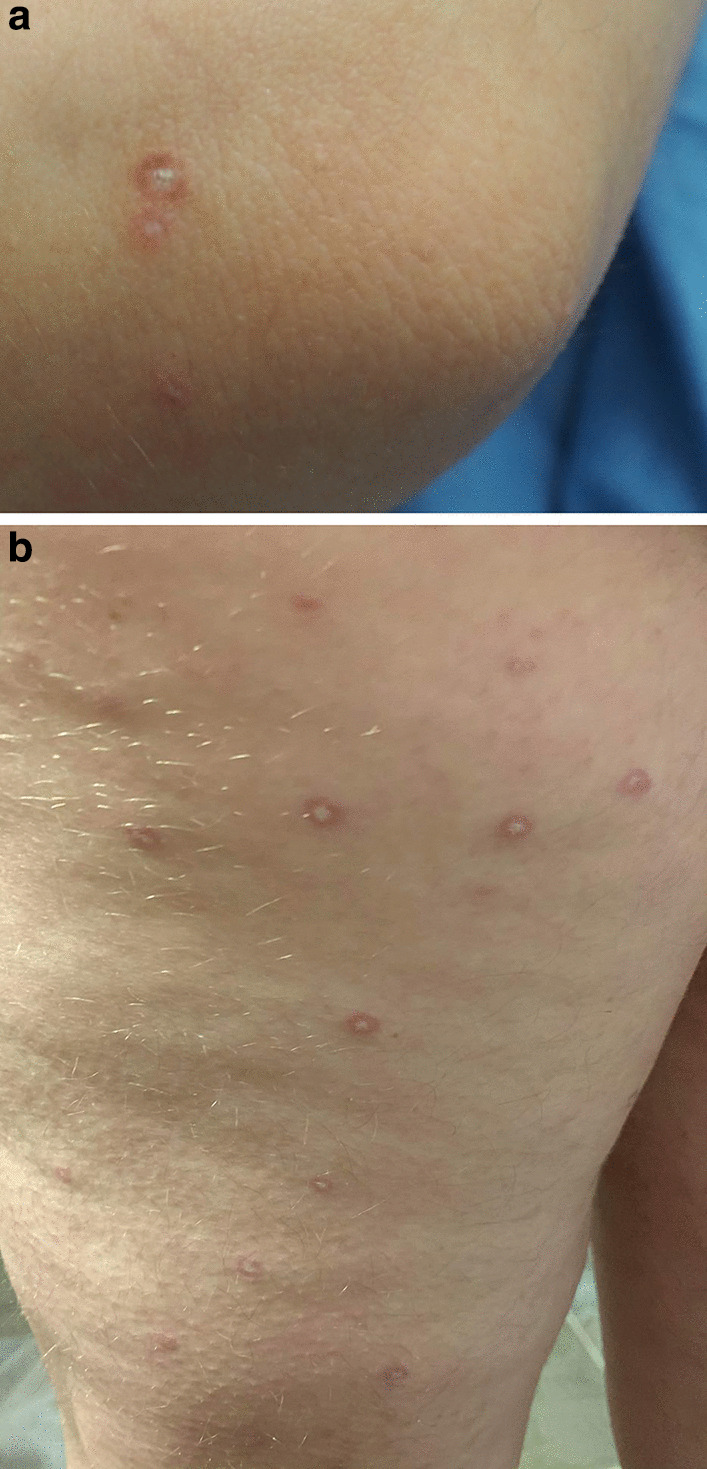
Clinical images of patient with atrophic papulosis. Porcelain white atrophic papules with a partially-blanchable red ring of are seen on the elbow (**a**) and lateral thigh (**b**)

Degos disease initially appears as small erythematous papules, approximately 0.5–1 cm in diameter [1] (Fig. 1a, b). Subsequently, the centre depresses to form a pathognomonic, porcelain-white atrophic papule with an erythematous, telangiectatic rim [1]. These lesions are usually found on the trunk and upper extremities [7, 8], but other locations such as palms, soles, scalp, and face are infrequently involved [1]. The first cutaneous lesions can appear at any age; however, most commonly occur between ages 20 and 50 [7]. The histologic findings classically involve wedge-shaped tissue necrosis due to thrombotic occlusion of small arteries deep in the corium [9, 10]; however these are not always present, as a review of 9 cases by Su et al. demonstrated infarct and wedge-shaped necrosis only in one biopsy [11]. The diagnosis of atrophic papulosis is, therefore, made clinically and supported by histologic findings.

MAP has been shown in the literature to primarily cause infarcts of the gastrointestinal (GI) system and central nervous system, as well as other organs such as the ocular, pulmonary, cardiovascular and renal systems [12, 13]. Bowel perforation and/or thrombosis or hemorrhage of cerebral arteries lead to death in 60% of cases; in the case of bowel vasculopathy, disease appears to progress from the serosal vessels [14]. Based on the only prospective 5-year study (N = 39) and reported case literature, atrophic papulosis most frequently presents cutaneously, with approximately 30% risk of subsequent internal organ involvement [2, 15]. In that cohort, they found that malignant disease was less likely to develop as time passed, with the probability of benign disease approaching 97% at 7 years [2]. The last large review of the case literature was performed in 1989 by Burg et al. (N = 103 cases) [9].

The aim of this systematic review of case and observational studies is two-fold: (1) to provide an update of the demographic and clinical features of patients with BAP and MAP and the therapies implemented, and (2) to identify clinical and laboratory factors associated with the development of MAP, in order to further aid in disease stratification and prognostication in patients with atrophic papulosis.

## Methods

The systematic review protocol is registered in PROSPERO (ID: CRD42020182930, https://www.crd.york.ac.uk/prospero/display_record.php?RecordID=182930). Additionally, we followed the Preferred Reporting Items for Systematic Reviews and Meta-Analyses (PRISMA) statement for our systematic review.

### Study identification and screening

Embase and MEDLINE in OVID database were systematically searched from inception to April 29^th^, 2020, the date of last search, using variations of the following search keywords: “Degos Disease”, “Köhlmeier-Degos”, “Atrophic Papulosis”, “Malignant Atrophic Papulosis” or “Benign Atrophic Papulosis” (complete search terms, Additional File 1). The title and abstract, and subsequently, the full texts of all exported articles were screened against the inclusion and exclusion criteria by two independent reviewers (J.D.L and M.S). The disagreements between the two reviewers were resolved through involvement of a third reviewer (D.C.).

### Inclusion criteria

Case reports, case series, observational cohort studies (controlled and uncontrolled), case control studies and randomized control trials conducted on patients with BAP/MAP were considered for inclusion. Eligibility for case inclusion required (i) diagnosis by a dermatologist and (ii) ≥ 1-year follow-up since onset of characteristic lesions (with the exception of cases deceased from systemic MAP before this timepoint). One year was selected as a minimal follow-up period given previous studies demonstrating a mean MAP survival of this duration [2]. The articles inaccessible through researchers’ affiliated institution or not available in the English language were excluded. If otherwise eligible studies were not diagnosed by a dermatologist but had sufficient information to support a diagnosis of Degos upon independent review, they were included.

### Data extraction

Abstract screening and data extraction were independently conducted by three authors (J.D.L, M.S, and O.M.S). Disagreements were resolved by discussion between the three authors and a fourth reviewer (D.C). Data extracted included study information (publication year and country, study design, number of cases), demographic data (age, sex), family history of autoimmune disease or Degos disease (y/n), primary or secondary Degos disease (secondary to systemic autoimmune disease), atypical location of lesions (facial or orogenital, y/n), generalized or localized (generalized defined as ≥ 3 body sites), duration since onset of cutaneous lesions, time to systemic involvement from onset of cutaneous lesions, survival, follow-up time from diagnosis, abnormal lab results (anti-nuclear antibody [ANA], antiphospholipid antibody [APLA], erythrocyte sedimentation rate/c-reactive protein [ESR/CRP]), treatments used, and organ involvement.

### Data analysis

We reported demographic and clinical features of patients with primary and secondary Degos descriptively, in relative and absolute terms, for our cohort of published cases. We used logistic multivariable regression to evaluate the association of multiple independent demographic and clinical exposure variables (age < 18, primary disease [y/n], atypical location [y/n], sex, elevated ESR or CRP [y/n], positive ANA [y/n]) with development of MAP, reported by odds ratio (OR) with 95% confidence intervals (CI). Cases with incompletely reported exposure (independent) variables were not included in the regression. Additionally, in cases of MAP that reported mortality, time to systemic disease and survival were depicted by Kaplan–Meier curves. Cases that did not report mortality were assumed to survive until last reported encounter, and cases that incompletely reported time to diagnosis or survival were excluded. No meta-analysis was planned.

### Quality assessment

The quality of evidence was assessed by J.D.L., M.S., and O.M.S using a modified version of the risk of bias tool by Murad et al. [16]. No studies were excluded based on quality.

## Results

### Search results

**Fig. 2:**
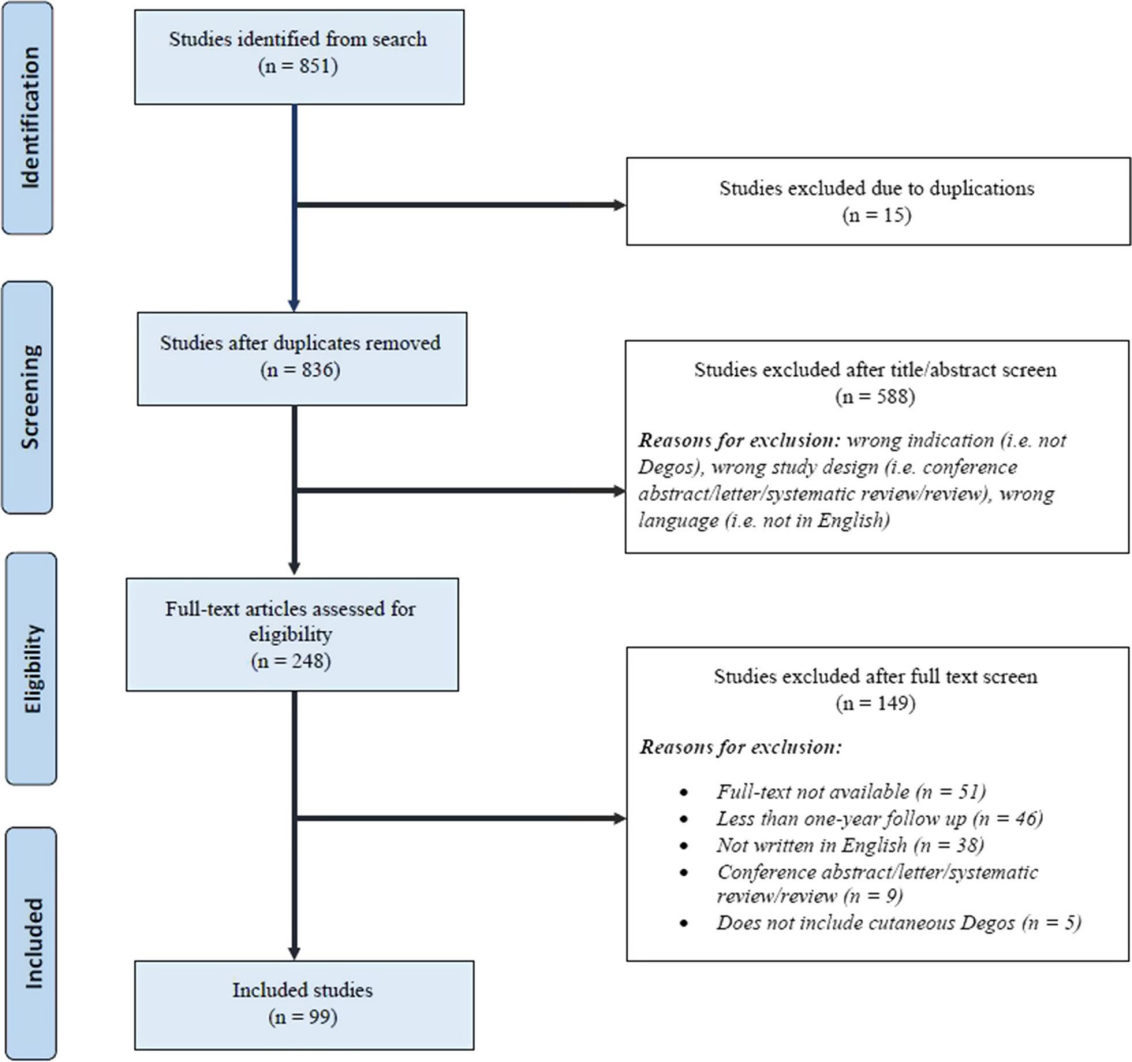
Preferred Reporting Items for Systematic Reviews and Meta-Analyses (PRISMA) flow diagram for identifying cases of Degos disease

A total of 851 studies were identified. Of these, 15 articles were excluded due to duplications, leaving 836 articles for title and abstract screening (Fig. 2). A total of 588 studies were excluded as they did not meet eligibility criteria, leaving 248 studies to be analyzed for full-text review. Reasons for exclusion were unavailable publications, less than one year follow-up, not English, non-primary article, and not Degos disease. Following the full-text review, a total of 99 studies were included for analysis (Fig. 2). All included studies were either case series or case reports.

### Demographics, clinical features, and treatments

From 99 included studies, we identified 105 patients with Degos disease, of which 63.8% had MAP and 36.2% had BAP (Table 1). Overall, the mean age of onset was 35 years (range: 0–75), with a slight female to male preponderance (F:M, 1.2:1). There was a higher incidence of females with BAP (68.4%, 26/38), but this did not meet significance by univariable regression (*p* = 0.0729, n = 105). Familial occurrence was 22.7% (n = 5/22, 83 NR) for both BAP and MAP patients. Patients with MAP had a described overall mortality rate of 71.6% (48/67), although in the absence of reported death, survival was assumed. The mean follow-up time was 2.55 years overall (SD 3.01), with BAP patients followed for longer on average (3.36 years, SD = 3.53) (Table 1).

**Table 1:** Demographics, clinical features, and treatments for patients with Degos disease

	All Patients	MAP	BAP
Number of patients	105	67	38
Male/female (ratio)	47/58 (1:1.2)	35/32 (1.1:1)	12/26 (1:2.2)
Mean age of cutaneous onset (stdev)	35 (0–75)	35 (0–75)	35 (0.2–63)
Familial occurrence, n (%)	5/22 (22.7)	2/14 (14.3)	3/8 (37.5)
Mortality, n (%)	N/A	48/67 (71.6%)	N/A
Mean follow up time of study	2.55 (3.01)	2.20 (2.69)	3.36 (3.53)
Distribution of lesions, n (%)			
Generalized	95/104 (91.3)	61/66 (92.4)	34/38 (89.5)
Localized	9/104 (8.7)	5/66 (7.6)	4/38 (10.5)
Primary or secondary Degos, n (%)			
Primary	94/105 (89.5)	63/67 (94)	31/38 (81.6)
Secondary	11/105 (10.5)	4/67 (6.0)	7/38 (18.4)
Dermatomyositis	2/105 (1.9)	0/67 (0)	2/38 (5.3)
Relapsing polychondritis	1/105 (1)	0/67 (0)	1/38 (2.6)
Rheumatoid arthritis	1/105 (1)	1/67 (1.5)	0/38 (0)
Systemic lupus erythematosus	8/105 (7.6)	2/67 (3)	6/38 (15.8)
Treatments, n (%)			
Anticoagulant	20/93 (21.5)	19/63 (30.2)	1/30 (3.3)
Antiplatelet (Single)	29/93 (31.2)	21/63 (33.3)	8/30 (26.7)
Antiplatelet (Dual)	19/93 (20.4)	14/63 (22.2)	5/30 (16.7)
Biologics			
Eculizumab	8/93 (8.6)	8/63 (12.7)	0/30 (0)
Infliximab	3/93 (3.2)	3/63 (4.8)	0/30 (0)
Natalizumab	1/93 (1.1)	1/63 (1.6)	0/30 (0)
Rituximab	2/93 (2.2)	2/63 (3.17)	0/30 (0)
Corticosteroids (topical and oral)	39/93 (41.2)	31/63 (49.2)	8/30 (26.7)
Immunomodulators			
Aminosalicylates	1/93 (1.1)	1/63 (1.6)	0/30 (0)
Azathioprine	2/93 (2.2)	1/63 (1.6)	1/30 (3.3)
Cyclosporin A	3/93 (3.2)	2/63 (3.2)	1/30 (3.3)
IVIg	13/93 (14)	11/63 (17.5)	2/30 (6.6)
Methotrexate	4/93 (4.3)	2/63 (3.2)	2/30 (6.6)
Tacrolimus	2/93 (2.2)	1/63 (1.6)	1/30 (3.3)
Thalidomide	1/93 (1.1)	1/63 (1.6)	0/30 (0)
Prostaglandin	2/93 (2.2)	1/63 (1.6)	1/30 (3.3)
Steroid sparing agents			
Cyclosporine	2/93 (2.2)	2/63 (3.2)	0/30 (0)
Dapsone	2/93 (2.2)	1/63 (1.6)	1/30 (3.3)
Others			
Cyclophosphamide	7/93 (7.5)	7/63 (11.1)	0/30 (0)
Pentoxifylline	9/93 (9.7)	6/63 (9.52)	3/30 (10)
No Treatment	7/93 (7.5)	1/63 (1.6)	6/30 (20)
Not Reported	12/93 (12.9)	4/63 (6.4)	8/30 (26.7)
Abnormal labs, n (%)			
ESR/CRP	14/76 (18.4)	13/48 (27.1)	1/28 (3.6)
ANA	10/75 (13.3)	4/46 (8.7)	6/29 (20.1)
Patient geographic distribution, n (%)			
Asia	33/105 (31.4)	23/67 (34.3)	10/38 (26.3)
North America	28/105 (26.7)	17/67 (25.4)	11/38 (28.9)
South America	2/105 (1.9)	2/67 (3)	0/38 (0)
Europe	38/105 (36.2)	22/67 (32.8)	16/38 (42.1)
Africa	0/105 (0)	0/67 (0)	0/38 (0)
Australia	4/105 (3.8)	3/67 (4.5)	1/38 (2.6)

MAP, malignant atrophic papulosis; BAP, benign atrophic papulosis; N/A, not applicable

The vast majority of patients (91.3%, 95/104) had lesions that were generalized on the trunk and extremities, and only 8.7% (n = 9/104) of patients were described with localized disease. The main treatments used were single or dual antiplatelet agents (51.6%, 48/93), followed by topical or oral corticosteroids (41.9%, 39/93,), anticoagulants (21.5%, 20/93), intravenous immunoglobulin (IVIg) (14%, 13/93), and eculizumab (8.6%, 8/93). Almost half of BAP patients were not treated or treatments were not reported (47%, 14/30); of the remainder, most used topical and oral corticosteroids (27%, 8/30) and/or antiplatelet agents (43%, 13/30).

### MAP organ involvement and survival

**Fig. 3:**
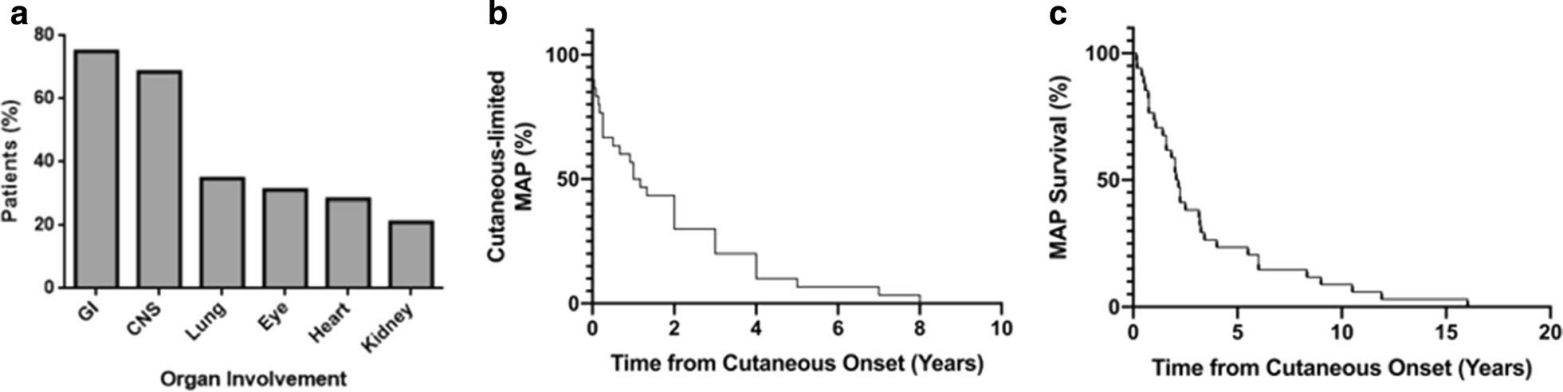
MAP survival and organ involvement. **a** MAP organ involvement predominately affects the gastrointestinal (GI) tract and central nervous system (CNS) (75% and 68.5% respectively). **b** Time to systemic involvement from first instance of cutaneous onset, 50% of patients had systemic involvement after 1 year (n = 30). **c** MAP survival from cutaneous onset, 53% of patients died by 2 years in cases that reported mortality with timeline data (n = 34)

Amongst 67 patients with MAP, systemic involvement predominately affected the GI tract (75%, 48/64, 3 NR) and central nervous system (CNS) (68.5%, 37/54, 13 NR); these were followed by lung (34.8%, 16/46, 21 NR), eye (31.1%, 14/45, 22 NR), heart (28.3%, 13/46, 21 NR), and kidney (21.1%, 8/38, 29 NR) (Fig. 3a). Among the cases of MAP with reported timeline data, the median survival of patients from cutaneous onset was 2.15 years (range = 0.17 to 16.04, n = 34) (Fig. 3b). In the subset of cases with time to systemic involvement data, the median duration was 1.09 years (range = 0 to 8 years, n = 30) (Fig. 3c).

### Factors associated with MAP development

Using multivariable logistic regression, we found that either or both elevated ESR and CRP were predictive of systemic involvement and distinguished MAP from BAP (OR 21.3, CI 2.62 to > 100) (Fig. 3c). Whereas elevated ESR or CRP was found in 27.1% (13/48) of patients with MAP, only 1 of 28 patients with BAP and ESR or CRP testing had elevations in both (Table 1). The calculated specificity and sensitivity are 96% and 27%, respectively. Furthermore, patients with Degos secondary to an autoimmune connective tissue disease (AiCTD) were less likely to develop MAP (OR 0.084, 95% CI 0.004 to 0.74) (Table 2). There were no significant associations been MAP and age < 18, sex, atypical site involvement (palmoplantar, orofacial, genital), or a positive ANA and/or hypocomplementemia (C3/C4). There were too few cases (4/75) with a measured anti-phospholipid antibody screen to power statistical analysis, and this was therefore omitted from our regression.

**Table 2:** Demographic, clinical and laboratory factors associated with the development of MAP (n = 75)

Independent variable	Odds ratio	95% CI	*p* value
Female	0.77	0.27 to 2.36	0.361
Comorbid AiCTD	**0.084**	**0.004 to 0.73**	**0.048**
Age < 18 years	0.91	0.23 to 3.7	0.0486
High ANA and/or low C3/C4	2.86	0.59 to 19.0	0.895
Atypical distribution	1.75	0.17 to 43.8	0.223
Elevated ESR and/or CRP	**21.3**	**2.62 to > 100**	**0.023**

Multivariable logistic regression of 75 cases demonstrates that Degos secondary to AiCTD is less frequently associated with development of MAP (OR = 0.08, CI 0.004 to 0.73). Elevations in either of ESR or CRP were associated with MAP (OR = 21.3, CI 2.62 to > 100). There were no associations been MAP and pediatric patients, sex, atypical distribution, or a positive anti-nuclear antibody and/or hypocomplementemia

## Discussion

Our systematic review of the literature identified 99 studies, comprising 105 patients with Degos disease for descriptive and quantitative analysis. Overall, 79.8% (79/99) studies were published after the year 1990 and time of last large review by Burg et al. [9] In our cohort of published cases, the disease predominantly occurred at the fourth decade of life, with systemic manifestations occurring in 63.8% of reported patients. The prognosis of MAP in our systematic cohort of published cases was poor, with an overall reported mortality rate of 71.6% (48/67, NR = 19) and a median survival of 2.15 years (range = 0.17 to 16.04, n = 34), from cutaneous onset. This is in keeping with the previously reported prospective cohort by Theodoridis et al., which found a median of 1 year for the development of systemic signs from the first occurrence of cutaneous lesions and median survival of only 0.9 years from the development of systemic disease (total 1.9 years from cutaneous onset, n = 11) [2].

Similar to Theodoridis et al., we found that there was no sex-associated prognosis by univariable or multivariable regression [2]. The slightly higher female preponderance for BAP (68.4%, 26/38) in our cohort may be related to the association found between Degos secondary to AiCTD and BAP and female predilection in SLE. As with previous studies [2, 9]. the GI tract and CNS were the extracutaneous systems with the highest reported involvement (75% and 68.5% respectively); we also noted the relatively high incidence of pulmonary (34.8%), ocular (31.1%), cardiac (28.3%), and renal (21.1%) involvement.

Unique to this systematic review is our utilization of multivariable regression to evaluate associations with MAP. We found that elevations in ESR or CRP may be a predictor of systemic involvement and may be an additional tool to assist in identifying the presence of systemic involvement. Although ESR/CRP are non-specific markers of inflammation [17] in the setting of Degos and in the absence of other causative conditions, they may infer systemic disease. This finding may have important implications for screening, since the bowel involvement in MAP is known to be primarily serosal, with poor visualization on both MRI and endoscopic imaging, and often requiring laparoscopic visualization for identification [18]. While ESR and CRP were found to be specific in this clinical context (96%), it is also crucial to highlight the poor test sensitivity (27%) and negative predictive value (60%) of these tests. Furthermore, one of this review’s authors is currently managing a patient with laparoscopically proven MAP, who has had normal ESR and CRPs and caution against ruling out systemic disease with this insensitive screen alone.

We conversely found that patients with Degos secondary to a systemic AiCTD were less likely to have MAP. The inverse correlation with MAP (OR 0.084, 95% CI 0.004 to 0.73) may suggest that AiCTD patients were incorrectly diagnosed as having MAP due to similar cutaneous lesions (such as discoid lupus or cutaneous vasculopathy from mixed connective tissue disease). Non-exclusively, it may also suggest that Degos associated with AiCTD has a different pathogenesis and natural history than primary idiopathic atrophic papulosis. The etiology of isolated MAP has been suspected to be mediated by complement; Magro et al. found extensive deposits of C5b-9 in the cutaneous vasculature, positing that C5 inhibition could be therapeutic. Eculizumab, a complement C5 inhibitor used for the treatment of paroxysmal nocturnal hemoglobinuria, has demonstrated some efficacy in delaying disease progression, with the addition of treponistil becoming the mainstay of therapy for patients who develop systemic disease [19, 20].

Further investigations are undoubtedly required to better understand the pathogenesis of Degos in order to effectively implement durable life-sustaining treatments. A critical first step is establishing how BAP differs from MAP, both from an epidemiological and biological perspective. This systematic review provides further insight into that differentiation and supports the use of ESR and CRP, amongst additional clinical and laboratory parameters, in the investigation and surveillance of a patient with undifferentiated atrophic papulosis.

### Limitations

Our study was primarily limited by its design as a SR of published literature comprising of case studies and series. While this design allowed for the inclusion of enough cases to power a quantitative analysis in an orphan disease, the possibility of publication bias of early poor outcomes is high, given the limited follow-up time in each published report and events such as mortality prompting publication. Furthermore, the evolution of BAP to MAP may also be underestimated due to the variable and limited follow-up times. Given that we did not capture symptomatic vs. asymptomatic patients or stratify severity among the cases with systemic involvement, it is unclear when ESR or CRP becomes sensitive for disease in MAP. Some studies did not completely report relevant clinical and laboratory details, such as total investigations performed and follow-up time prior to publication; these studies were included for descriptive analysis but not for Kaplan Meier or multivariable regression analysis, as relevant.

## Conclusions

Clinicians should be aware of the use of ESR/CRP in identifying evolution of BAP to MAP, as well as the association of secondary Degos with a more benign disease course. Further prospective studies are required to understand both the evolution and the pathogenesis of this disease to inform treatment and long term management.

## Supplementary Information

**Additional file 1.** Supplemental Tables.

## Data Availability

The authors declare that all data supporting the findings of this study are available within the article and its supplementary information files.
