# Supplementary material for: Clinical and laboratory prognosticators of atrophic papulosis (Degos disease): a systematic review
Source: Orphanet J Rare Dis. 2021 May 6;16:203. doi: 10.1186/s13023-021-01819-z (PMC8101154; doi:10.1186/s13023-021-01819-z)
Supplement: Supplementary file 1 — Additional file 1. Supplemental Tables. [file 13023_2021_1819_MOESM1_ESM.docx]

**SUPPLEMENTAL TABLES**

**Table S1.** Quality assessment for case reports/series. Adapted from Murad et al [[16](#_ENREF_16)].

| **Study number** | **Study** | **Ascertainment** | **Causality** | **Reporting** | **Total Score** |
| --- | --- | --- | --- | --- | --- |
|  |  | Biopsy diagnosis or dermatology diagnosis | Alternative diagnoses ruled out | Cases that reported demographics, temporality, basis of diagnosis |  |
| 1 | Winkelmann et al., 1963 [[21](#_ENREF_21)] | Yes | No | Yes | 2 |
| 2 | Feuerman, 1966 [[22](#_ENREF_22)] | Yes | No | Yes | 2 |
| 3 | Howard et al., 1968 [[23](#_ENREF_23)] | Yes | Yes | Yes | 3 |
| 4 | Durie et al., 1969 [[24](#_ENREF_24)] | Yes | Yes | Yes | 3 |
| 5 | Hall-Smith, 1969 [[25](#_ENREF_25)] | Yes | Yes | Yes | 3 |
| 6 | Salomon et al., 1971 [[26](#_ENREF_26)] | Yes | Yes | Yes | 3 |
| 7 | Jensen, 1972 [[27](#_ENREF_27)] | Yes | Yes | Yes | 3 |
| 8 | Muller & Landry, 1976 [[28](#_ENREF_28)] | Yes | Yes | Yes | 3 |
| 9 | Black et al., 1976 [[29](#_ENREF_29)] | Yes | Yes | Yes | 3 |
| 10 | McFarland et al., 1978 [[30](#_ENREF_30)] | Yes | Yes | Yes | 3 |
| 11 | Pierce & Smith, 1978 [[31](#_ENREF_31)] | Yes | No | Yes | 2 |
| 12 | Stahl et al., 1978 [[32](#_ENREF_32)] | Yes | Yes | Yes | 3 |
| 13 | Olmos et al., 1979 [[33](#_ENREF_33)] | Yes | No | Yes | 2 |
| 14 | Dastur et al., 1981 [[34](#_ENREF_34)] | Yes | Yes | Yes | 3 |
| 15 | Lee et al., 1984 [[35](#_ENREF_35)] | Yes | No | Yes | 2 |
| 16 | Englert et al., 1984 [[36](#_ENREF_36)] | Yes | Yes | Yes | 3 |
| 17 | Kisch et al., 1984 [[5](#_ENREF_5)] | Yes | Yes | Yes | 3 |
| 18 | Habbema et al., 1986 [[37](#_ENREF_37)] | Yes | No | Yes | 2 |
| 19 | Schade et al., 1987 [[38](#_ENREF_38)] | Yes | No | Yes | 2 |
| 20 | Rosemberg et al., 1988 [[39](#_ENREF_39)] | Yes | Yes | Yes | 3 |
| 21 | Burrow et al., 1991 [[40](#_ENREF_40)] | Yes | Yes | Yes | 3 |
| 22 | Demitsu et al., 1992 [[41](#_ENREF_41)] | Yes | Yes | Yes | 3 |
| 23 | Leslie et al., 1993 [[42](#_ENREF_42)] | Yes | Yes | Yes | 3 |
| 24 | Vázquez-Doval et al., 1993 [[7](#_ENREF_7)] | Yes | Yes | Yes | 3 |
| 25 | Mauad et al., 1996 [[43](#_ENREF_43)] | Yes | Yes | Yes | 3 |
| 26 | Török et al., 1996 [[44](#_ENREF_44)] | Yes | Yes | Yes | 3 |
| 27 | Tsao et al., 1997 [[45](#_ENREF_45)] | Yes | No | Yes | 2 |
| 28 | Willa-Craps et al., 1997 [[46](#_ENREF_46)] | Yes | Yes | Yes | 3 |
| 29 | Fruhwirth et al., 1997 [[47](#_ENREF_47)] | Yes | Yes | Yes | 3 |
| 30 | Demitsu et al., 1997 [[48](#_ENREF_48)] | Yes | Yes | Yes | 3 |
| 31 | Requena et al., 1998 [[49](#_ENREF_49)] | Yes | Yes | Yes | 3 |
| 32 | Farrell et al., 1998 [[50](#_ENREF_50)] | Yes | Yes | Yes | 3 |
| 33 | al-Smadi et al., 2000 [[51](#_ENREF_51)] | Yes | No | Yes | 2 |
| 34 | Güven et al., 2000 [[52](#_ENREF_52)] | Yes | No | Yes | 2 |
| 35 | Egan & Lessell, 2000 [[53](#_ENREF_53)] | Yes | No | Yes | 2 |
| 36 | Harvell et al., 2001 [[54](#_ENREF_54)] | Yes | Yes | Yes | 3 |
| 37 | Beales, 2001 [[15](#_ENREF_15)] | Yes | Yes | Yes | 3 |
| 38 | Torrelo et al., 2002 [[55](#_ENREF_55)] | Yes | Yes | Yes | 3 |
| 39 | Ojeda Cuchillero et al., 2003 [[56](#_ENREF_56)] | Yes | Yes | Yes | 3 |
| 40 | Kanekura et al., 2003 [[57](#_ENREF_57)] | Yes | Yes | Yes | 3 |
| 41 | Thomas et al., 2003 [[13](#_ENREF_13)] | Yes | No | Yes | 2 |
| 42 | Kocheril et al., 2004 [[58](#_ENREF_58)] | Yes | Yes | Yes | 3 |
| 43 | High et al., 2004 [[59](#_ENREF_59)] | Yes | No | Yes | 2 |
| 44 | Coskun et al., 2004 [[60](#_ENREF_60)] | Yes | Yes | Yes | 3 |
| 45 | Loewe et al., 2005 [[61](#_ENREF_61)] | Yes | Yes | Yes | 3 |
| 46 | Fernández-Pérez et al., 2005 [[62](#_ENREF_62)] | Yes | Yes | Yes | 3 |
| 47 | Zamiri et al., 2005 [[63](#_ENREF_63)] | Yes | Yes | Yes | 3 |
| 48 | Aydogan et al., 2005 [[12](#_ENREF_12)] | Yes | Yes | Yes | 3 |
| 49 | Amato et al., 2005 [[64](#_ENREF_64)] | Yes | Yes | Yes | 3 |
| 50 | Matsuura et al., 2006 [[65](#_ENREF_65)] | Yes | Yes | Yes | 3 |
| 51 | Ullah-Khan & Rafiq S, 2006 [[66](#_ENREF_66)] | Yes | No | Yes | 2 |
| 52 | Zhu et al., 2007 [[67](#_ENREF_67)] | Yes | Yes | Yes | 3 |
| 53 | Wilson et al., 2007 [[68](#_ENREF_68)] | Yes | Yes | Yes | 3 |
| 54 | De Breucker et al., 2008 [[69](#_ENREF_69)] | Yes | Yes | Yes | 3 |
| 55 | Kim et al., 2008 [[70](#_ENREF_70)] | Yes | Yes | Yes | 3 |
| 56 | Yamaguchi et al., 2008 [[71](#_ENREF_71)] | Yes | Yes | Yes | 3 |
| 57 | Amaravadi et al., 2008 [[72](#_ENREF_72)] | Yes | No | Yes | 2 |
| 58 | Rizos et al., 2008 [[73](#_ENREF_73)] | Yes | No | Yes | 2 |
| 59 | Subramaniam et al., 2008 [[74](#_ENREF_74)] | Yes | Yes | Yes | 3 |
| 60 | Nikoo et al., 2009 [[75](#_ENREF_75)] | Yes | No | Yes | 2 |
| 61 | Moss et al., 2009 [[76](#_ENREF_76)] | Yes | Yes | Yes | 3 |
| 62 | Chung et al., 2009 [[77](#_ENREF_77)] | Yes | Yes | No | 2 |
| 63 | Ortiz et al., 2010 [[78](#_ENREF_78)] | Yes | Yes | Yes | 3 |
| 64 | Mutizwa et al., 2010 [[79](#_ENREF_79)] | Yes | Yes | Yes | 3 |
| 65 | Zheng et al., 2010 [[80](#_ENREF_80)] | Yes | No | Yes | 2 |
| 66 | Gupta et al., 2011 [[81](#_ENREF_81)] | Yes | Yes | Yes | 3 |
| 67 | Yeo et al., 2011 [[82](#_ENREF_82)] | Yes | Yes | Yes | 3 |
| 68 | Gutiérrez-Pascual et al., 2011 [[83](#_ENREF_83)] | Yes | Yes | Yes | 3 |
| 69 | Ali et al., 2011 [[84](#_ENREF_84)] | Yes | Yes | Yes | 3 |
| 70 | Ahmadi et al., 2011 [[85](#_ENREF_85)] | Yes | Yes | Yes | 3 |
| 71 | Pati et al., 2011 [[86](#_ENREF_86)] | Yes | Yes | Yes | 3 |
| 72 | Magro et al., 2013 [[87](#_ENREF_87)] | Yes | No | Yes | 2 |
| 73 | Yamaguchi et al., 2013 [[88](#_ENREF_88)] | Yes | Yes | Yes | 3 |
| 74 | Yeung et al., 2013 [[89](#_ENREF_89)] | Yes | Yes | Yes | 3 |
| 75 | Shapiro et al., 2013 [[20](#_ENREF_20)] | Yes | Yes | Yes | 3 |
| 76 | Guo et al., 2014 [[90](#_ENREF_90)] | Yes | Yes | Yes | 3 |
| 77 | Zhu et al., 2014 [[91](#_ENREF_91)] | Yes | Yes | Yes | 3 |
| 78 | Zaharia et al., 2014 [[92](#_ENREF_92)] | Yes | Yes | Yes | 3 |
| 79 | Liu et al., 2014 [[93](#_ENREF_93)] | Yes | Yes | Yes | 3 |
| 80 | Umemura et al., 2015 [[94](#_ENREF_94)] | Yes | Yes | Yes | 3 |
| 81 | Kim et al., 2015 [[95](#_ENREF_95)] | Yes | Yes | Yes | 3 |
| 82 | Su et al., 2015 [[96](#_ENREF_96)] | Yes | Yes | Yes | 3 |
| 83 | Oliver et al., 2016 [[97](#_ENREF_97)] | Yes | Yes | Yes | 3 |
| 84 | Flühler et al., 2016 [[98](#_ENREF_98)] | Yes | Yes | Yes | 3 |
| 85 | Gmuca et al., 2016 [[99](#_ENREF_99)] | Yes | No | Yes | 2 |
| 86 | Hiernickel et al., 2016 [[100](#_ENREF_100)] | Yes | Yes | Yes | 3 |
| 87 | Calderón-Castrat et al., 2017 [[101](#_ENREF_101)] | Yes | Yes | Yes | 3 |
| 88 | Jang et al., 2017 [[102](#_ENREF_102)] | Yes | Yes | Yes | 3 |
| 89 | Zouboulis et al., 2017 [[103](#_ENREF_103)] | Yes | Yes | Yes | 3 |
| 90 | Ye et al., 2018 [[104](#_ENREF_104)] | Yes | Yes | Yes | 3 |
| 91 | Hu et al., 2018 [[105](#_ENREF_105)] | Yes | Yes | Yes | 3 |
| 92 | Huang et al., 2018 [[106](#_ENREF_106)] | Yes | Yes | Yes | 3 |
| 93 | Kim & Motaparthi, 2018 [[107](#_ENREF_107)] | Yes | No | Yes | 2 |
| 94 | Churruca-Grijelmo et al., 2018 [[108](#_ENREF_108)] | Yes | No | Yes | 2 |
| 95 | Goswami et al., 2019 [[109](#_ENREF_109)] | Yes | No | Yes | 2 |
| 96 | Saracino et al., 2019 [[110](#_ENREF_110)] | Yes | No | Yes | 2 |
| 97 | Stavorn & Chanprapaph, 2019 [[111](#_ENREF_111)] | Yes | Yes | Yes | 3 |
| 98 | Razanamahery et al., 2020 [[112](#_ENREF_112)] | Yes | Yes | Yes | 3 |
| 99 | Mareschal et al., 2020 [[113](#_ENREF_113)] | Yes | Yes | Yes | 3 |

**Table S2.** Study and patient characteristics with associated risk of bias.

| **Patient number** | **Study, year** | **Age of lesion onset** | **Sex** | **MAP/BAP** | **Primary or secondary** | **Localized or generalized** | **Location of lesion** | **APLA** | **ANA/C3** | **CRP/ESR** | **Duration since onset (years)** | **Survival from diagnosis (years)** | **Follow-up time from initial presentation (years)** | **Time to systemic involvement (years)** |
| --- | --- | --- | --- | --- | --- | --- | --- | --- | --- | --- | --- | --- | --- | --- |
| 1 | Winkelmann et al., 1963 [[21](#_ENREF_21)] | 19 | M | MAP | Primary | Generalized | Trunk and extremities | NR | NR | N | 1.75 | 1.75 | NR | 0.75 |
| 2 | Winkelmann et al., 1963 [[21](#_ENREF_21)] | 33 | M | MAP | Primary | Generalized | Trunk and extremities | NR | NR | N | 3 | 3 | NR | 2 |
| 3 | Feuerman, 1966 [[22](#_ENREF_22)] | 43 | M | BAP | Primary | Generalized | Trunk, extremities (proximal and flexor) | NR | NR | NR | 3 | NR | NR | N/A |
| 4 | Howard et al., 1968 [[23](#_ENREF_23)] | 21 | F | MAP | Primary | Generalized | Face | N | N | N | 0.08 | NR | 1.58 | 0.08 |
| 5 | Durie et al., 1969 [[24](#_ENREF_24)] | 34 | M | MAP | Primary | Generalized | Face | N | A | A | 0.33 | 0.25 | 0.25 | 0 |
| 6 | Hall-Smith, 1969 [[25](#_ENREF_25)] | 16 | M | MAP | Primary | Generalized | Face | N | N | N | 0.5 | 0.25 | 0.25 | 0.25 |
| 7 | Hall-Smith, 1969 [[25](#_ENREF_25)] | 45 | F | BAP | Primary | Generalized | Right arm, abdomen, legs | N | N | N | 4 | NR | 4 | N/A |
| 8 | Salomon et al., 1971 [[26](#_ENREF_26)] | 47.5 | F | BAP | Primary | Generalized | Face | NR | N | NR | 0 | NR | 2 | N/A |
| 9 | Jensen, 1972 [[27](#_ENREF_27)] | 50 | F | MAP | Primary | Generalized | Trunk, proximal limbs | N | N | A | 1 | NR | 0.5 | 1 |
| 10 | Muller & Landry, 1976 [[28](#_ENREF_28)] | 63 | F | BAP | Primary | Generalized | Trunk and extremities | N | N | N | 2 | NR | 3 | N/A |
| 11 | Muller & Landry, 1976 [[28](#_ENREF_28)] | 24 | F | BAP | Primary | Generalized | right arm, right axilla, trunk | N | N | N | 1 | NR | NR | N/A |
| 12 | Black et al., 1976 [[29](#_ENREF_29)] | 39 | F | BAP | Secondary | Generalized | Face, Upper Extremities | A | A | N | NR | NR | NR | NR |
| 13 | Black et al., 1976 [[29](#_ENREF_29)] | 37 | F | BAP | Secondary | Generalized | Trunk, Face, Upper | N | A | N | NR | NR | NR | N/A |
| 14 | McFarland et al., 1978 [[30](#_ENREF_30)] | 47 | M | MAP | Primary | Localized | Trunk | N | N | N |  | 1.08 | 1.08 | 0 |
| 15 | Pierce & Smith, 1978 [[31](#_ENREF_31)] | 21 | F | MAP | Primary | Generalized | Trunk and extremities | NR | NR | NR | 0.92 | 11 | 11.08 | 0.08 |
| 16 | Stahl et al., 1978 [[32](#_ENREF_32)] | 30 | M | BAP | Primary | Generalized | Face | N | N | N | 0 | NR | 10 | N/A |
| 17 | Olmos et al., 1979 [[33](#_ENREF_33)] | 42 | F | BAP | Primary | Generalized | Body | NR | NR | NR | 1.67 | NR | NR | N/A |
| 18 | Dastur et al., 1981 [[34](#_ENREF_34)] | 42 | M | MAP | Primary | Generalized | Trunk and extremities | N | N | N | 0.58 | 0.17 | 0.17 | 0.25 |
| 19 | Lee et al., 1984 [[35](#_ENREF_35)] | 42 | F | BAP | Primary | Generalized | Upper and lower extremities, trunk, and the soles and dorsa of the feet | NR | NR | NR | 7 | NR | 4 | N/A |
| 20 | Englert et al., 1984 [[36](#_ENREF_36)] | 36 | F | MAP | Primary | NR | NR | N | N | A | 1 | 1 | 1 | 2 |
| 21 | Kisch et al., 1984 [[5](#_ENREF_5)] | 39 | M | BAP | Primary | Generalized | Trunk and extremities | N | N | N | 1 | NR | 1 | N/A |
| 22 | Habbema et al., 1986 [[37](#_ENREF_37)] | 56 | F | BAP | Primary | Generalized | NR | NR | NR | NR | 1 | NR | NR | NR |
| 23 | Schade et al., 1987 [[38](#_ENREF_38)] | 7 | M | MAP | Primary | Generalized | NR | NR | NR | NR | 0 | NR | 7 | 7 |
| 24 | Rosemberg et al., 1988 [[39](#_ENREF_39)] | 9 | F | MAP | Primary | Generalized | Face | N | N | N | 5 | 1 | 1 | 5 |
| 25 | Burrow et al., 1991 [[40](#_ENREF_40)] | 22 | M | MAP | Primary | Generalized | Trunk and extremities | N | N | N | 0.08 | 0.75 | NR | 0.17 |
| 26 | Demitsu et al., 1992 [[41](#_ENREF_41)] | 28 | M | BAP | Primary | Generalized | Trunk, extremities | N | N | N | 1.08 | NR | 1.58 | N/A |
| 27 | Leslie et al., 1993 [[42](#_ENREF_42)] | 54 | F | MAP | Primary | Generalized | Trunk and extremities | N | N | N | 7 | NR | 7 | 4 |
| 28 | Vázquez-Doval et al., 1993 [[7](#_ENREF_7)] | 36 | M | MAP | Primary | Generalized | Trunk and extremities | N | N | N | 0 | 2 | 2 | 2 |
| 29 | Vázquez-Doval et al., 1993 [[7](#_ENREF_7)] | 17 | M | BAP | Primary | Generalized | Trunk and extremities | N | N | N | 0 | NR | 8 | N/A |
| 30 | Mauad et al., 1996 [[43](#_ENREF_43)] | 22 | M | MAP | Primary | Generalized | Body | A | N | N | NR | 6 | 6 | NR |
| 31 | Török et al., 1996 [[44](#_ENREF_44)] | 40 | F | BAP | Secondary | Generalized | Face | N | A | N | 0 | 12 | 10 | N/A; N/A |
| 32 | Tsao et al., 1997 [[45](#_ENREF_45)] | 26 | F | BAP | Primary | Generalized | Arms and thighs bilaterally | NR | NR | NR | 0.5 | NR | 1 | N/A |
| 33 | Willa-Craps et al., 1997 [[46](#_ENREF_46)] | 33 | F | BAP | Primary | Localized | Trunk | N | NR | NR | 1.33 | NR | 0.33 | N/A |
| 34 | Fruhwirth et al., 1997 [[47](#_ENREF_47)] | 51 | M | MAP | Primary | Generalized | Trunk and upper arm | N | N | N | NR | 5.42 | 5.42 | NR |
| 35 | Demitsu et al., 1997 [[48](#_ENREF_48)] | 52 | F | BAP | Primary | Generalized | Trunk and abdomen | N | N | A | 1.5 | NR | 2 | N/A |
| 36 | Requena et al., 1998 [[49](#_ENREF_49)] | 58 | M | BAP | Primary | Generalized | Trunk, extensors of upper/ lower extremities, dorsum of hands, palms | N | N | N | 0.17 | NR | 2.33 | N/A |
| 37 | Farrell et al., 1998 [[50](#_ENREF_50)] | 44 | F | MAP | Primary | Generalized | Trunk, proximal limbs | A | N | N | 0.25 | NR | 4 | N/A |
| 38 | Al-Smadi et al., 2000 [[51](#_ENREF_51)] | 20 | M | BAP | Primary | Generalized | Trunk and extremities | NR | NR | NR | 4 | NR | 4 | N/A |
| 39 | Güven et al., 2000 [[52](#_ENREF_52)] | 43 | F | MAP | Primary | Generalized | Submammary area, legs, feet, arms | NR | NR | NR | 4.5 | NR | 4.5 | 3.5 |
| 40 | Egan & Lessell, 2000 [[53](#_ENREF_53)] | 42 | M | MAP | Primary | Generalized | Hands, forearms, chest | NR | NR | NR | 0 | 2.5 | 2.5 | 1 |
| 41 | Harvell et al., 2001 [[54](#_ENREF_54)] | 51 | M | BAP | Primary | Generalized | Chest, back, arms | N | N | N | 2 | NR | NR | N/A |
| 42 | Beales, 2001 [[15](#_ENREF_15)] | 48 | M | MAP | Primary | Generalized | Trunk and extremities | NR | NR | NR | NR | 0.17 | 0.17 | 0.08 |
| 43 | Torrelo et al., 2002 [[55](#_ENREF_55)] | 0.2 | F | BAP | Primary | Generalized | Trunk and extremities | N | N | N | 0.42 | NR | 1 | N/A |
| 44 | Ojeda Cuchillero et al., 2003 [[56](#_ENREF_56)] | 24 | M | BAP | Primary | Generalized | Trunk, arms, lower limbs, palms | N | N | N | 2.5 | NR | 8 | N/A |
| 45 | Kanekura et al., 2003 [[57](#_ENREF_57)] | 24 | F | BAP | Primary | Generalized | Trunk and legs | N | N | N | 0.33 | NR | 2 | N/A |
| 46 | Thomas et al., 2003 [[13](#_ENREF_13)] | 16 | M | MAP | Primary | Generalized | Trunk and extremities | NR | NR | NR | 3 | 0.19 | 0.19 | 0.04 |
| 47 | Kocheril et al., 2004 [[58](#_ENREF_58)] | 49 | F | MAP | Primary | Generalized | Oral, back, extremities, trunk | N | N | N | 0 | 1.83 | 1.83 | 0.92 |
| 48 | High et al., 2004 [[59](#_ENREF_59)] | 50 | F | MAP | Primary | Generalized | Extremities, trunk | N | N | N | 6 | 4.5 | 4.5 | 8 |
| 49 | Coskun et al., 2004 [[60](#_ENREF_60)] | 14 | M | BAP | Primary | Localized | Back | N | N | N | 20 | NR | NR | N/A |
| 50 | Loewe et al., 2005 [[61](#_ENREF_61)] | 27 | M | BAP | Primary | Localized | Chest | N | N | N | 6 | NR | NR | N/A |
| 51 | Fernández-Pérez et al., 2005 [[62](#_ENREF_62)] | 38 | M | MAP | Secondary (SLE) | Localized | Trunk | N | N | N | 5 | 0.17 | 0.17 | NR |
| 52 | Zamiri et al., 2005 [[63](#_ENREF_63)] | 24 | F | BAP | Primary | Generalized | Trunk, limbs | N | N | N | 8 | NR | 0.17 | N/A |
| 53 | Aydogan et al., 2005 [[12](#_ENREF_12)] | 42 | M | MAP | Primary | Generalized | Face | A | N | A | 1 | 0.08 | 0.08 | NR |
| 54 | Amato et al., 2005 [[64](#_ENREF_64)] | 26 | F | MAP | Primary | Generalized | Trunk and extremities | NR | NR | NR | 3 |  | N/A | 3 |
| 55 | Matsuura et al., 2006 [[65](#_ENREF_65)] | 58 | F | MAP | Primary | Generalized | Trunk and proximal extremities | N | N | N | NR | 0.67 | 0.67 | 0 |
| 56 | Ullah-Khan & Rafiq S, 2006 [[66](#_ENREF_66)] | 49 | F | BAP | Primary | Generalized | Trunk and extremities | N | N | N | 1 | NR | 1 | NR |
| 57 | Zhu et al., 2007 [[67](#_ENREF_67)] | 37 | F | MAP | Primary | Generalized | Face | N | N | A | 1 | NR | 1.92 | 0.75 |
| 58 | Wilson et al., 2007 [[68](#_ENREF_68)] | 10 | F | BAP | Primary | Generalized | Thighs, arms | N | N | N | 6 | NR | 1.33 | N/A |
| 59 | De Breucker et al., 2008 [[69](#_ENREF_69)] | 56 | M | MAP | Primary | Generalized | Trunk, upper extremities | NR | A | N | 4 | 4.33 | 4.33 | 4 |
| 60 | Kim et al., 2008 [[70](#_ENREF_70)] | 57 | F | MAP | Primary | Generalized | Trunk and lower extremities | A | NR | NR | 2 | 0.25 | 2.25 | 1.17 |
| 61 | Yamaguchi et al., 2008 [[71](#_ENREF_71)] | 25 | F | MAP | Primary | Generalized | Legs, trunks, arms | N | N | A | 0.67 | 0.33 | 0.33 | 0.5 |
| 62 | Amaravadi et al., 2008 [[72](#_ENREF_72)] | 40 | F | MAP | Primary | Generalized | Legs, arms, trunk | NR | NR | NR | NR | 1.42 | 1.42 | 1.33 |
| 63 | Rizos et al., 2008 [[73](#_ENREF_73)] | 41 | F | MAP | Primary | Generalized | Trunk and extremities | NR | NR | NR | 1.5 | 0.08 | 0.08 | 1.33 |
| 64 | Subramaniam et al., 2008 [[74](#_ENREF_74)] | 75 | F | MAP | Primary | Generalized | Trunk and extremities | N | N | A | 8 | 8.04 | 8.04 | 4 |
| 65 | Nikoo et al., 2009 [[75](#_ENREF_75)] | 40 | M | MAP | Primary | Generalized | Genitals, Face | NR | NR | NR | 7 | 2 | 2 | 7 |
| 66 | Moss et al., 2009 [[76](#_ENREF_76)] | 0.5 | M | MAP | Primary | Generalized | Genitals, Trunk | N | N | N | 0.02 | 0.15 | 0.15 | 0.02 |
| 67 | Chung et al., 2009 [[77](#_ENREF_77)] | 43 | F | MAP | Primary | Generalized | Trunk and extremities | NR | N | A | NR | 1.5 | 1.5 | 0 |
| 68 | Ortiz et al., 2010 [[78](#_ENREF_78)] | 31 | F | BAP | Secondary (SLE) | Generalized | Upper and lower extremities | N | A | N | N/A | NR | 6 | N/A |
| 69 | Mutizwa et al., 2010 [[79](#_ENREF_79)] | 38 | M | BAP | Secondary (SLE) | Localized | Back | N | A | N | 3 | NR | 1.33 | NR |
| 70 | Zheng et al., 2010 [[80](#_ENREF_80)] | 36 | F | MAP | Primary | Generalized | Trunk, limbs | N | N | N | 1.33 | 0.25 | 0.33 | 1 |
| 71 | Gupta et al., 2011 [[81](#_ENREF_81)] | 50 | M | MAP | Secondary (dermatomyositis-like picture) | Generalized | Face | NR | NR | NR | 0.67 | 0.06 | 0.083 | 0.67 |
| 72 | Yeo et al., 2011 [[82](#_ENREF_82)] | 0 | F | MAP | Primary | Generalized | Trunk, extremities | N | N | N | 3 | 3 | 3 | 0.15 |
| 73 | Gutiérrez-Pascual et al., 2011 [[83](#_ENREF_83)] | 0.25 | F | MAP | Primary | Generalized | Face | N | N | N | 4 | NR | 4 | 1.25 |
| 74 | Ali et al., 2011 [[84](#_ENREF_84)] | 45 | M | MAP | Primary | Generalized | Genitals, Trunk | N | N | A | 3 | 0.42 | 0.42 | 3 |
| 75 | Ahmadi et al., 2011 [[85](#_ENREF_85)] | 13 | M | MAP | Primary | Generalized | Trunk, lower extremities | N | N | N | 2 | 0.25 | 0.25 | 2 |
| 76 | Pati et al., 2011 [[86](#_ENREF_86)] | 41 | M | MAP | Primary | Localized | Primarily trunk | N | N | N | 0.33 | NR | 0.58 | 0.33 |
| 77 | Magro et al., 2013 [[87](#_ENREF_87)] | 46 | M | MAP | Primary | Generalized | Trunk, upper extremities, neck, shoulders | N | NR | NR | 2 | NR | 2.42 | 2 |
| 78 | Yamaguchi et al., 2013 [[88](#_ENREF_88)] | 66 | M | MAP | Primary | Generalized | Trunk and extremities, spared face, palms, soles | N | N | A | NR | 0.67 | 0.67 | NR |
| 79 | Yeung et al., 2013 [[89](#_ENREF_89)] | 50 | M | MAP | Primary | Generalized | Trunk and extremities | N | N | N | 2 | 2 | 2 | 0.25 |
| 80 | Shapiro et al., 2013 [[20](#_ENREF_20)] | 36 | F | BAP | Secondary | Generalized | Trunk and extremities | NR | A | N | 1 | NR | 5 | N/A |
| 81 | Shapiro et al., 2013 [[20](#_ENREF_20)] | 17 | M | MAP | Primary | Generalized | Trunk and extremities | NR | NR | NR | 2 | NR | 1.5 | NR |
| 82 | Guo et al., 2014 [[90](#_ENREF_90)] | 5 | M | MAP | Primary | Generalized | Face | N | N | N | 4 | NR | 1.42 | 3 |
| 83 | Zhu et al., 2014 [[91](#_ENREF_91)] | 46 | F | MAP | Primary | Generalized | Trunk, extremities | N | N | N | 7.83 | NR | 7.83 | 0.17 |
| 84 | Zaharia et al., 2014 [[92](#_ENREF_92)] | 38 | M | MAP | Primary | Generalized | Trunk, limbs | NR | N | N | 3 | NR | 3 | NR |
| 85 | Liu et al., 2014 [[93](#_ENREF_93)] | 39 | F | BAP | Primary | Generalized | Trunk and extremities | N | N | N | 2 | NR | 2 | N/A |
| 86 | Umemura et al., 2015 [[94](#_ENREF_94)] | 68 | M | MAP | Primary | Generalized | Trunk and extremities, spared face, palms, soles | N | N | A | NR | 0.67 | 0.67 | NR |
| 87 | Kim et al., 2015 [[95](#_ENREF_95)] | 25 | F | BAP | Primary | Generalized | Trunk and extremities | N | N | N | 20 | NR | 0.33 | N/A |
| 88 | Su et al., 2015 [[96](#_ENREF_96)] | 41 | F | MAP | Primary | Generalized | Face | N | A | N | 0.17 | 2 | 2 | N/A |
| 89 | Oliver et al., 2016 [[97](#_ENREF_97)] | 50 | F | MAP | Primary | Generalized | Extremities, chest, back, abdomen | N | N | N | 5 | NR | NR | 1 |
| 90 | Flühler et al., 2016 [[98](#_ENREF_98)] | 47 | F | MAP | Primary | Generalized | Face | N | N | N | 0 | 0.42 | 0.42 | 0 |
| 91 | Gmuca et al., 2016 [[99](#_ENREF_99)] | 4 | M | MAP | Primary | Localized | Foot | NR | NR | NR | 0.17 | 0.02 | 0.02 | 0.17 |
| 92 | Hiernickel et al., 2016 [[100](#_ENREF_100)] | 26 | F | MAP | Primary | Generalized | Trunk and extremities | N | N | N | 1 | 0.42 | 0.42 | NR |
| 93 | Calderón-Castrat et al., 2017 [[101](#_ENREF_101)] | 0.4 | F | BAP | Primary | Generalized | Trunk and extremities | NR | N | N | 0 | NR | 1.17 | N/A |
| 94 | Jang et al., 2017 [[102](#_ENREF_102)] | 29 | F | MAP | Secondary (SLE) | Generalized | Fingers, knees | N | A | N | 2 | NR | 0.17 | N/A |
| 95 | Zouboulis et al., 2017 [[103](#_ENREF_103)] | 54 | F | BAP | Primary | Generalized | Torso, extremities | N | N | N | 2 | NR | 16 | N/A |
| 96 | Ye et al., 2018 [[104](#_ENREF_104)] | 21 | F | MAP | Secondary (RA) | Localized | Lower limbs | NR | NR | NR | 11 | NR | 11 | 0 |
| 97 | Hu et al., 2018 [[105](#_ENREF_105)] | 30 | F | MAP | Primary | Generalized | Trunk, extremities, sparing palms, face, genitals | N | N | A | 3 | 0.25 | 0.25 | 3 |
| 98 | Huang et al., 2018 [[106](#_ENREF_106)] | 4 | F | MAP | Primary | Generalized | Trunk, thighs | N | N | N | 4 | 1.5 | 1.67 | 4 |
| 99 | Kim & Motaparthi, 2018 [[107](#_ENREF_107)] | 50 | F | BAP | Primary | Generalized | Trunk, extremities | NR | NR | NR | 0.5 | NR | 1.5 | N/A |
| 100 | Churruca-Grijelmo et al., 2018 [[108](#_ENREF_108)] | 46 | F | MAP | Primary | Generalized | Trunk and extremities | NR | NR | NR | NR | 6.25 | 6.25 | 6 |
| 101 | Goswami et al., 2019 [[109](#_ENREF_109)] | 24 | M | MAP | Primary | Generalized | Hands, feet, back | NR | NR | NR | 2 | 0.06 | 0.06 | 2 |
| 102 | Saracino et al., 2019 [[110](#_ENREF_110)] | 44 | M | MAP | Primary | Generalized | Trunk and extremities | NR | NR | NR | 0.25 | 0.27 | 0.27 | NR |
| 103 | Stavorn & Chanprapaph, 2019 [[111](#_ENREF_111)] | 59 | F | BAP | Secondary | Generalized | Face | NR | A | NR | 0 | NR | 4 | N/A |
| 104 | Razanamahery et al., 2020 [[112](#_ENREF_112)] | 28 | M | MAP | Primary | Generalized | Trunk, upper limb, back | N | N | A | 3.08 | 0.08 | 0.08 | 3 |
| 105 | Mareschal et al., 2020 [[113](#_ENREF_113)] | 35 | M | MAP | Primary | Generalized | Trunk, upper extremities | N | A | N | 3 | NR | 0.5 | 3 |

**Abbreviations:** N, normal; NR, not reported; N/A; not applicable; A, abnormal lab result (above or below normal range); SLE, systemic lupus erythematosus; RA, rheumatoid arthritis; MAP, malignant atrophic papulosis; BAP, benign atrophic papulosis.
